# Influence of molecular genetics in Vogt-Koyanagi-Harada disease

**DOI:** 10.1186/s12348-014-0020-1

**Published:** 2014-07-22

**Authors:** Joanne YW Ng, Fiona OJ Luk, Timothy YY Lai, Chi-Pui Pang

**Affiliations:** 1 Department of Ophthalmology and Visual Sciences, The Chinese University of Hong Kong, 3/F Hong Kong Eye Hospital, 147K Argyle Street, Kowloon ᅟ, Hong Kong

**Keywords:** Vogt-Koyanagi-Harada disease, Genetics, Human leukocyte antigen, Single-nucleotide polymorphisms, Interleukins

## Abstract

Vogt-Koyanagi-Harada (VKH) disease is a systemic autoimmune disorder against melanocytes. Recent studies have identified multiple genetic factors that might be associated with the pathogenesis of VKH disease. We performed an electronic database search of PubMed, MEDLINE, and EMBASE, and all relevant papers published up to 13 June 2014 were reviewed. A total of 1,031 publications including articles relevant to the genetics of VKH disease and the references of these articles were reviewed. The review identified a number of genetic factors which might be involved in the pathogenesis of VKH disease, some of which may alter the clinical course of VKH disease. Genes which might be involved in the pathogenesis of VKH disease included genes expressing HLA, complement factor H, interleukins, cytotoxic T-lymphocyte antigen 4 (CTLA-4), killer cell immunoglobulin-like receptors (KIR), programmed cell death 1 (PDCD1), protein tyrosine phosphatase non-receptor 22 (PTPN22), osteopontin, tumor necrosis factor alpha-induced protein 3 (TNFAIP3), macrophage migration inhibitory factor (MIF), and other immune response genes. Further studies to explore the correlation among different genotypes and phenotypes of VKH disease will be useful to shed light on the pathogenesis of uveitis in VKH disease and may facilitate the development of new treatment modalities of uveitis in VKH disease.

## Review

### Introduction

Vogt-Koyanagi-Harada (VKH) disease is a systemic autoimmune disorder against melanocytes and affects the eyes, skin, ears, and meninges [[1]-[4]]. VKH disease usually occurs more frequently in females with a female-to-male ratio of approximately 2:1 and is one of the top three leading causes of uveitis in China [[4]-[6]]. It is characterized by bilateral granulomatous uveitis and can be classified into four stages: prodromal, uveitic, convalescence, and chronic recurrent [[7]]. In the prodromal and uveitic phases, there are neurological and auditory manifestations, and integumentary findings usually appear in the convalescent and chronic recurrent phases of the disease [[2]]. Together with the absence of past ocular surgery, the ocular, neurological, auditory, and integumentary findings help to establish the diagnosis of VKH disease [[8]]. Treatment for VKH disease generally involves aggressive systemic corticosteroid treatment during the acute stage to reduce the risk of permanent visual loss [[9]-[12]]. Corticosteroid treatment should be tapered off slowly and maintained for at least 6 months as early withdrawal of oral corticosteroid has been found to be a significant risk factor for recurrence of VKH disease and might lead to worse visual outcome [[13]].

The precise etiology of VKH disease is unclear, and it has been postulated that pathogenesis involves an autoimmune process directed against melanocytes triggered by an infectious agent in genetically susceptible individuals [[1]], leading to the loss of melanocytes and subsequent depigmentation [[14],[15]]. The potential role of infection in the pathogenesis of VKH disease is supported by the presence of Epstein-Barr virus DNA in vitreous aspiration of a patient with VKH disease [[16]], and cross-reactions between melanocyte peptides and cytomegalovirus envelope glycoprotein resulting in melanocyte proliferation [[17]]. However, a causative association between viral agents and VKH disease has not yet been established. The autoimmune response against melanocytes is complex and involves innate, humoral, and cellular immunity. CD4+ and CD8+ lymphocytes, as well as Th17 and regulatory T cells, have all been implicated in the pathogenesis [[18]]. High titres of IgG against KU-MEL-1, an antigen expressed by human melanocytes, have been detected in patients with VKH disease [[19]]. Innate immunity may also play an important role in the pathogenesis of VKH disease as evidenced by the presence of histiocytic and multinucleated giant cell infiltrates in enucleated eyes with VKH disease [[14]]. In combination with environmental factors, genetic factors are of paramount importance in various immune processes in VKH disease. This review aims to provide an overview on the role of genetics in the development of VKH disease and the clinical importance and implications of genetics in VKH disease.

### Human leukocyte antigen genes

The major histocompatibility complex (MHC) in human is known as human leukocyte antigen (HLA). The MHC region in human is located at 6p21.3 on the short arm of chromosome 6, containing more than 200 genes and over 40 of which are HLA genes [[20]]. The HLA genes encode a wide variety of antigen-presenting molecules on cell surface and proteins with immunological function. The HLA genes involved in immune function are classified into three classes [[20],[21]]: MHC class I region contains genes that encode HLA-A, HLA-B, and HLA-C which form complexes with peptides of antigens and then export these antigens to the cell surface; MHC class II region contains genes encoding HLA-DM, HLA-DO, HLA-DP, HLA-DQ, and HLA-DR which are present in antigen-presenting cells and bind extracellular antigens in order to present them to T cells; and MHC class III region contains genes that play important roles in immune response including several components of complement (C2, C4, and factor B), tumor necrosis factor (tumor necrosis factor-alpha, lymphotoxin alpha, and lymphotoxin beta), and heat shock protein. Both MHC class I and class II are expressed on the cell surface to present antigens to the T lymphocytes in order to initiate immune response.

Among the MHC class II genes, more than 1,500 different alleles have been identified, and studies have provided evidence that MHC class II are associated with various autoimmune diseases [[21]-[23]]. MHC class II genes are expressed on activated T cells and antigen-presenting cells including dendritic cells, macrophages, and B cells. Both the α- and β-chain genes are present in each subregion of DR, DP, and DQ and form a specific MHC class II molecule [[20]]. A number of HLA genotypes have been strongly linked with VKH disease, and these include HLA-DR and HLA-DQ (Tables 1 and 2).

**Table 1:** Major HLA genotypes associated with VKH disease

**HLA genotype association**	**Major findings**	**Race**	**References**
HLA-DQA1*0301	100% of 57 VKH patients vs. 67.2% of 122 control subjects	Japanese	Islam et al. [[24]]
HLA-DQB1*0604	0% of 57 VKH patients vs. 15.6% of 122 control subjects, i.e., may be protective against VKH disease	Japanese	Islam et al. [[24]]
HLA-DRB1*0404	25% of 76 VKH patients vs. 9.8% of 256 healthy individuals	Mexican Mestizo	Alaez et al. [[40]]
HLA-DRB1*0405	95% of 40 VKH patients vs. 58.2% of DR4-positive healthy controls	Japanese	Shindo et al. [[28]]
	54.1% of 37 VKH patients have HLA-DRB1*0405 with a relative risk of 11.76 over the general population	Highly admixed Brazilian	Goldberg et al. [[34]]
	82.3% of 18 VKH patients vs. 9.3% of 128 control subjects	Korean	Kim et al. [[35]]
	36.6% of 30 VKH patients vs. 6.9% of 29 control subjects	Saudi Arabian	Iqniebi et al. [[36]]
	13.2% of 76 VKH patients vs. 0.4% of 256 healthy individuals	Mexican Mestizo	Alaez et al. [[40]]

**Table 2:** Major HLA serotypes associated with VKH disease

**HLA serotype association**	**Major findings**	**Race**	**References**
HLA-DQ4	83% of 57 VKH patients vs. 32% of 461 control subjects	Japanese	Islam et al. [[24]]
HLA-DQw7	59.4% of 32 VKH patients vs. 36.5% of 52 control subjects	Japanese	Zhang et al. [[25]]
HLA-DR1	36% of 25 VKH patients vs. 9% of 217 control subjects	Hispanic	Weisz et el [[30]]
HLA-DR4	75% of 32 VKH patients vs. 23.1% of 52 control subjects	Chinese	Zhang et al. [[25]]
	93% of 57 VKH patients vs. 43% of 461 control subjects	Japanese	Islam et al. [[24]]
	56% of 25 VKH patients vs. 29% of 217 control subjects	Hispanic	Weisz et el [[30]]
HLA-DR53	98.2% of 57 VKH patients vs. 67.5% of 461 control subjects	Japanese	Islam et al. [[24]]

#### HLA-DQ

Islam et al. conducted a study in Japanese VKH patients and found that HLA-DQ4 and HLA-DQA1*0301 were present in 83% and 100% of VKH patients, respectively, compared with 32% and 67%, respectively, in control subjects, with HLA-DQA1*0301/-DR4 showing the highest risk ratio (Table 1) [[24]]. The study also showed that HLA-DQB1*0604 could not be detected in any VKH patient, and therefore, it might offer some protection against VKH disease by modifying the pathogenesis of VKH [[24]].

#### HLA-DR

A large number of studies have shown that VKH disease is strongly associated with various HLA-DR alleles (Table 1) [[25]]. HLA-DR4 of MHC class II was first found to be associated with VKH disease in Japanese in 1976 [[26]]. Since then, studies have demonstrated that HLA-DR4 is strongly associated with VKH patients of different ethnic groups (Tables 1 and 2) including North Americans [[27]], Chinese [[25]], Japanese [[28],[29]], Hispanics [[30]-[32]], Italians [[33]], ethnically heterogeneous Brazilian [[34]], Koreans [[35]], and Saudi Arabians [[36]].

Over two decades ago, Goldberg et al. demonstrated that HLA-DRB1*0405 was a major allele responsible for susceptibility to develop VKH disease in Brazilian patients which is a highly admixed population [[34]]. In the study [[34]], the diverse alleles initially suggested to be associated with VKH disease occurred independently, strengthening the significance of HLA-DRB1*0405 in the pathogenesis of VKH. The frequency of HLA-DRB1*0405 was also significantly higher in Japanese patients with prolonged type of VKH disease [[37]]. Further studies have demonstrated that patients with VKH disease who were HLA-DRB1*0405-positive recognized a greater range of melanocyte epitopes than HLA-matched control subjects [[38]], consolidating the influence of HLA-DRB1*0405 in affecting the susceptibility to VKH disease.

In addition to HLA-DRB1*0405 mentioned above, HLA-DRB1*0407 and HLA-DRB1*0410 alleles were also found to have increased frequency in Mestizo patients living in southern California [[32]]. It was also found that the frequency of HLA-DRB1*0410 was increased in Japanese patients with VKH disease [[24],[28],[39]]. Increased frequencies of HLA-DRB1*0405 and HLA-DRB1*0404 were identified in Mexican Mestizo VKH patients with odds ratios of 2.95 and 2.79, respectively [[40]]. The serotype HLA-DR1 has also been suspected as having a possible association with VKH disease [[30]]. However, alleleic analyses did not find a significant association between HLA-DRB1 alleles with VKH disease [[36]].

S57-LLEQRRAA (67-74) located in the third hypervariable region of the HLA-DRB1 molecule is a shared epitope of the two HLA-DRB1*0405 and HLA-DRB1*0410 alleles [[41]]. This shared epitope was found to be linked with VKH disease in Indian patients [[41]]. Results from alleleic and epitopic studies have demonstrated that epitopes may be involved in the immunopathogenesis of VKH disease. However, complex associations between different epitopes and VKH disease are expected due to the high variability of HLA and the differences in genetic profiles across different ethnic populations [[42],[43]].

Although multiple studies have shown that the genetic predisposition to VKH disease is contributed by multiple overlapping factors encoded by the HLA system, the strengths of association between HLA-DRB1*04 subtypes and VKH disease vary among different races, indicating that the pathogenesis of VKH may be multifactorial with additional genetic susceptibility and environmental causes [[32],[34]].

### Cytotoxic T-lymphocyte antigen 4 gene

Cytotoxic T-lymphocyte antigen 4 (*CTLA-4*) gene is located on chromosome 2q33 and is expressed in activated T cells and inhibits T cell activation by CD28 [[44]]. Over 100 single-nucleotide polymorphisms (SNPs) have been identified in *CTLA-4* gene regions, and some of these SNPs were found to be related to immune-mediated diseases including type 1 diabetes mellitus [[45]], multiple sclerosis [[46]], systemic lupus erythematosus (SLE) [[47]], rheumatoid arthritis [[48],[49]], Hashimoto's thyroiditis, and Graves' disease [[45]]. However, some of the studies showed conflicting results in the association of *CTLA-4* gene polymorphisms and autoimmune diseases [[50]-[52]].

A study of Chinese Han patients with VKH disease provided evidence that the G allele at SNP +49 of *CTLA-4* was associated with VKH disease, and the *CTLA-4* haplotype −1661A:−318C:+49G:CT60G was also found to confer risk of VKH disease [[53]]. The *CTLA-4* haplotype has been shown to be associated with other types of uveitis, and this can be due to differences in the pathogenesis mechanisms among different uveitis entities [[53]].

### Complement system

The complement system is an essential component of the primary immune system, and it plays a key role in regulating various immunological and inflammatory responses. It has been demonstrated that experimental autoimmune uveoretinitis (EAAU) can be caused by activation of the complement system, and depletion of the host's complement system can completely inhibit the EAAU [[54],[55]]. Yang et al. have shown that in Chinese females, the 184G rs800292 polymorphism of complement factor H (*CFH*) gene is a genetic risk marker of anterior uveitis [[56]]. Another study also showed that the carrier frequency of G allele of *CFH*-rs800292 is increased in patients with non-infectious intermediate and posterior uveitis [[57]]. However, so far, SNPs in *CFH* that are associated with VKH disease have not yet been discovered.

### Interleukin genes

Interleukins (ILs) are produced by leukocytes and are potent inflammatory mediators which are heavily involved in numerous immunological diseases [[58]-[60]] including diverse entities of uveitis [[59],[61]-[63]]. Studies have demonstrated that various interleukins may be associated with VKH disease (Table 3).

**Table 3:** Interleukin (IL) genes that have been suggested to be associated with VKH disease

**IL genes**	**Polymorphisms associated with VKH disease**	**Functional relevance**	**Race**	**References**
*IL-12B*	rs3212227	C allele of rs3212227 of *IL-12B* is a risk factor of VKH disease. IL-12B is suggested to enhance Th1 production which is involved in the pathogenesis of VKH disease.	Chinese Han	Li et al. [[67]]
*IL-17F*	rs763780	TT genotype of *IL-17F*-rs763780 predisposes susceptibility to VKH disease, whereas the C allele of rs763780 may have protective effects against VKH disease.	Chinese Han	Shu et al. [[69]]
*IL-23R*	None discovered yet.	IL-23 levels are increased in the serum of VKH patients with active uveitis. IL-23 enhances production of IL-27 by CD4^+^ T cells.	Chinese Han	Jiang et al. [[73]]
*IL-27*	None discovered yet.	IL-27 is suggested to promote Th17 production which is involved in the pathogenesis of VKH disease.	Chinese Han	Yang et al. [[57]]

#### Interleukin-12B gene

Interleukin-12 (IL-12) is critical in the differentiation of naïve T cells into Th1 cells [[64]] and was identified to be involved in the pathogenesis of Behcet's disease [[65],[66]]. Recently, C allele of rs3212227 of the *IL-12B* gene was shown to be a significant risk factor of VKH disease [[67]].

#### Interleukin-17 gene

Upregulation of interleukin-17 (IL-17) was identified to be associated with intraocular inflammation in patients of VKH disease and Behçet's disease [[68]]. It was also shown that rs763780 of *IL-17F* was associated with VKH disease in Chinese Han population, with the TT genotype increasing the susceptibility of VKH disease and the C allele of rs763780 being possibly protective against VKH disease [[69]]. However, since only two SNPs of the *IL-17* gene were tested in the study [[69]], further studies are needed to evaluate if the other polymorphisms of the *IL-17* gene are also associated with VKH disease.

#### Interleukin-23 receptor gene

Interleukin-23 (IL-23) was identified to be an important cytokine in the development of autoimmune diseases [[70],[71]]. It enhances the production of IL-17 by CD4^+^ T cells and contributes to the maintenance of various autoimmune diseases [[72]]. Previous study has found that the levels of IL-23 in the serum of VKH patients with active uveitis were significantly higher than those without active uveitis and normal controls [[68]]. However, no statistical significant association was found in four selected polymorphisms (rs17375018, rs7517847, rs11209032, and rs1343151) of interleukin-23 receptor (*IL23R*) gene in VKH patients from a Chinese Han population [[73]]. In view of the discrepancy between the SNP analysis and the current understanding in the functions of IL-23 in uveitis, more SNPs in the *IL23R* gene should be explored to investigate its association with VKH disease.

#### Interleukin-27 gene

Interleukin-27 (*IL-27*) is expressed in photoreceptors and retinal ganglion cells [[74]]. It enhances the differentiation of naïve T cells into Th1 cells but suppresses naïve T cells from differentiating into Th17 cells, resulting in mutual antagonism of Th1 and Th17 cells which are both involved in the pathogenesis of uveitis [[75],[76]]. Nonetheless, a previous study by Yang et al. showed no association between the rs4788084 SNP of *IL-27* and intermediate uveitis and posterior uveitis [[57]].

### Killer cell immunoglobulin-like receptor gene cluster

The killer cell immunoglobulin-like receptor (*KIR*) genes on chromosome 19 encode inhibitory (3DL1, 3DL2, 3DL3, 2DL1, 2DL2, 2DL3, and 2DL5) and activating (3DS1, 2DS1, 2DS2, 2DS3, 2DS4, 2DS5) receptors which are expressed on the majority of natural killer (NK) cells and a small proportion of T cells [[77]]. The inhibitory KIRs recognize distinct HLA class I molecules and stop the effector function of NK cells, thus offering protections to healthy cells. Expression of HLA class I molecules protect healthy cells from surveillance of NK cells [[78]].

*KIR2DS3* was found to be more frequent in VKH patients than in the control group in a study performed in Saudi Arabia [[79]]. Another study by Levinson et al. showed that the frequency of *KIR* gene cluster 3DS1-2DL5-2DS1-2DS5 was higher and increased the susceptibility to VKH disease in Japanese patients [[80]]. Therefore, it was suggested that the *KIR* genes that encode activating KIR receptors may increase the risk of VKH disease. On the other hand, KIR-HLA interactions might be involved in the protection against VKH disease and might possibly reduce the severity of VKH disease as KIR2DL2/2DL3+HLA-C1 was identified to have a higher frequency in the control group [[79]]. However, the direct role of NK cells in the pathogenesis of VKH disease has not yet been established.

### Programmed cell death 1 gene

Programmed cell death 1 (*PDCD1*) on chromosome 2q37 encodes programmed cell death 1 (PD-1) which induces apoptotic cell death of murine lymphoid cell lines *in vitro* [[81]]. It has been suggested to suppress the development of inflammatory helper T cells modulating the innate immune system [[82]]. *PDCD1* has been shown to be involved in a wide range of autoimmune diseases including Graves' disease [[83]], type I diabetes [[84]], ankylosing spondylitis [[85]], rheumatoid arthritis [[86],[87]], SLE [[88]-[91]], and multiple sclerosis [[92]]. However, several studies have shown contradictory results [[93]-[97]]. In Chinese Han population, the frequency of C allele in PD-1.5 was found to be significantly lower in VKH patients with poliosis or with dysacusis compared with control subjects, suggesting that PD-1.5 may influence the extraocular manifestations among VKH patients [[98]]. In the same study, no association was found among VKH disease and the SNPs PD-1.3 and PD-1.6 [[98]].

### Protein tyrosine phosphatase non-receptor 22 gene

Protein tyrosine phosphatase non-receptor 22 (PTPN22) influences the development and activation of lymphocytes, innate cell-mediated immune host defense, formation of tolerance, and regulation of the immune system [[99]]. *PTPN22* is located on chromosome 1p13.3 to 1p13.1 and encodes the lymphoid-specific phosphatase known as Lyp which plays an essential suppressive role in T cell activation [[100]]. Several studies have demonstrated that a SNP in *PTPN22*, R620W (rs2476601), predisposes individuals to various autoimmune diseases including insulin-dependent diabetes mellitus [[101]], rheumatoid arthritis [[102],[103]], Graves' disease [[104]], and SLE [[105]]. In a study of 67 Japanese patients with VKH disease [[106]], six SNPs in *PTPN22* (rs3811021, rs1217413, rs1237682, rs3761935, rs3789608, and rs2243471) were shown to have no significant association with VKH disease. However, in a study of 1,005 VKH patients and 2,010 healthy controls of Han Chinese population [[107]], significantly increased frequencies of rs2488457 CC genotype and C allele were found in VKH patients. Moreover, there was decreased frequency of rs2488457 GG genotype in patients with VKH disease. No significant association between T cell activation and rs2488457 genotype was observed [[107]]. It was proposed that a functional variant rs2488457 in *PTPN22* increases susceptibility to VKH disease via modulating the expression of *PTPN22*, production of interleukin 10 (IL-10) and proliferation of peripheral blood mononuclear cells [[107]].

### Osteopontin gene

The osteopontin (*OPN*) gene encodes osteopontin which is a pro-inflammatory cytokine involved in chronic inflammatory diseases [[108],[109]]. It was shown that serum levels of OPN were significantly higher in patients with active VKH disease than in patients with inactive VKH disease and in the control group [[110]]. In the same study [[110]], recombinant OPN was shown to induce a significant increase in the proliferation of CD4^+^ T cells and secretion of interferon gamma and IL-17 in patients with active VKH disease. The frequency of the TT genotype of OPN rs4754 was also shown to be positively correlated with VKH disease in Chinese Han population [[110]].

### Tumor necrosis factor, alpha-induced protein 3 gene

Tumor necrosis factor, alpha-induced protein 3 (*TNFAIP3*) gene encodes the A20 protein which is a cytoplasmic zinc finger protein. The A20 protein prevents the over-reaction of innate immune responses by suppressing TNF-induced signaling and negatively regulating nuclear factor kappa B (NF-κB) responses mediated by innate immune receptors including TNF receptor [[111],[112]]. *TNFAIP3* polymorphisms were found to predispose to autoimmune diseases including rheumatoid arthritis [[113],[114]], psoriasis [[115]], and SLE [[116],[117]]. A study by Li et al. in VKH patients has found that the rs9494885 TC genotype and C allele may confer a risk to VKH disease, while the rs9494885 TT genotype and T allele may protect against VKH disease [[118]].

### Macrophage migration inhibitory factor gene

The macrophage migration inhibitory factor (*MIF*) gene on chromosome 22q11.2 plays an important role in the level of MIF expression in macrophages and T cells, as well as release of other inflammatory cytokines [[119]]. SNPs in the *MIF* gene have been shown to be involved in a number of immunological diseases including psoriasis [[120]], SLE [[121]], ulcerative colitis [[122]], juvenile idiopathic arthritis [[123],[124]], and multiple sclerosis [[125]]. The levels of MIF were also found to be significantly higher in uveitis patients with VKH disease, sarcoidosis, and Behçet's disease [[126],[127]]. In a study by Zhang et al. [[128]], the frequencies of GG genotype and G allele of rs755622 in the *MIF* gene were found to be significantly lower in VKH patients than in controls. The frequency of T allele of rs2096525 was also significantly lower in patients with headache or vitiligo than in the control group [[128]]. Therefore, the GG genotype and G allele may be protective factors for VKH disease, while the T allele of rs2096525 may be a risk factor for non-ocular manifestation of VKH disease [[128]]. The study also demonstrated that the combined rs755622/rs2096525 CT haplotype confers an increased risk to VKH disease, whereas the GT haplotype reduces the susceptibility to VKH disease [[128]].

### Tyrosinase gene family

Tyrosinase gene family is specifically expressed in melanocytes, and it encodes the enzymes that forms melanin [[129]]. Mutations of tyrosinase gene family are related to depigmentation and developmental defects of the eye including oculocutaneous albinism type 1 (OCA1) and microphthalmia [[130]-[133]]. Lymphocytes of patients with VKH disease were shown to be reactive to peptides derived from tyrosinase family proteins, thus it is possible that tyrosinase and tyrosinase-related proteins could be the auto-antigens in VKH disease [[1]]. However, a study evaluating polymorphisms of microsatellites loci in tyrosinase gene family among Japanese VKH patients has failed to find a significant association with VKH disease [[134]].

### Interferon gamma gene

Previous studies have demonstrated elevated levels of interferon gamma in the aqueous humor and serum of patients with VKH disease [[135],[136]]. However, a study evaluating gene polymorphism of interferon gamma gene and VKH disease found no significant difference in the frequencies of alleles and genotypes of interferon gamma gene between VKH patients and healthy controls [[137]].

### NLR family, pyrin domain containing 1 gene

NLR family, pyrin domain containing 1 (*NLRP1*) gene on chromosome 17p13 is involved in the primary immune system and was shown to be associated with generalized vitiligo and other autoimmune diseases [[138]-[141]]. Since skin hypopigmentation in VKH disease resembles generalized vitiligo, *NLRP1* might be involved in the pathogenesis of VKH. However, a study by Horie et al. has shown that *NLRP1* gene polymorphisms related to vitiligo were not associated with the risks or clinical manifestations of VKH disease, and this suggests that the genetic and immune factors associated with VKH disease are likely to be distinct from generalized vitiligo [[142]].

### Toll-like receptor 9 gene

Toll-like receptor 9 (TLR9) recognizes unmethylated 2′-deoxyribo(cytidine-phosphate-guanosine) (CpG) dinucleotide motifs in viruses and is of importance in the immunological defense against viral infections [[143]]. *TLR9* has been postulated to be one of the candidate genes in the pathogenesis of VKH, but a study by Ito et al. has failed to find a significant association between *TLR9* polymorphisms and VKH disease [[144]].

### Transforming growth factor β receptor gene

Transforming growth factor β ((TGF-β) influences the differentiation of Th17 [[145]-[147]], and two SNPs of type III TGF-β receptor (rs1805110 and rs2489188) have been investigated to determine their association with VKH disease in Chinese Han population [[148]]. These two SNPs were shown not to be associated with VKH disease, whereas rs2489188 CC genotype of the type III TGF-β receptor was shown to be protective against Behcet's disease [[148]].

### Janus kinase 1 gene

Janus kinase 1 (*JAK1*) is an essential component of the human immune system which regulates cell differentiation of Th1 and Th17 [[149]]. A recent study suggested that three SNPs of the *JAK1* gene including GG genotype of rs310230, GG genotype of rs310236, and TT genotype of rs310241 were found to occur in lower frequencies in VKH patients than control subjects [[150]]. However, no significant link between the three SNPs and clinical manifestations of VKH disease was identified, and currently, there is no published data on the functional role of these SNPs of *JAK1* [[150]].

### Fibroblast growth factor receptor 1 oncogene partner and chemokine (C-C motif) receptor 6 genes

Both fibroblast growth factor receptor 1 oncogene partner (*FGFR1OP*) and chemokine receptor 6 (*CCR6*) are encoded by a linkage disequilibrium block located on chromosome 6q27 [[151]], and both have been identified to be associated with susceptibility of vitiligo. Moreover, the two genes have been implicated in the pathogenesis of autoimmune diseases as *CCR6* was found to be associated with Crohn's disease [[152]], ulcerative colitis [[153]], and rheumatoid arthritis [[154],[155]], while *FGFR1OP* was found to be related to Graves' disease [[156]] and myeloproliferative disorders [[157],[158]] and is involved in microtubule anchoring at the centrosome [[159]]. Two independent case-control cohorts of Chinese Han population have shown that the A allele of rs2301436 of *FGFR1OP* is associated with susceptibility to VKH, whereas no association was found for the four tested *CCR6* SNPs including rs3093024, rs6902119, rs3093023, and rs968334 [[160]].

### Limitations of genetic studies in VKH disease

Although genetic studies have contributed greatly in identifying mechanisms involved in the pathogenesis of VKH disease, they are not without any limitation. Firstly, there may be linkage disequilibrium in the genetic analysis, i.e., the occurrence of certain combinations of alleles or genetic markers in a population more frequently or less frequently than these would be expected from a totally random formation of haplotypes from alleles based on their original frequencies. Linkage equilibrium occurs when there is non-random correlation among neighboring alleles that descend from single ancestral chromosomes [[161]]. The genetic association found in the different studies may be in linkage disequilibrium with the causative locus, thereby leading to confounding of the results. Secondly, the effects of genotypes on phenotypes found in many of the research are not clearly known, and therefore, exactly how the SNPs affect the inflammatory proteins and their receptor functions remains uncertain. Therefore, whether the manipulations of these inflammatory proteins in patients with certain genotypes would be able to alter the course of VKH disease remain to be seen.

## Conclusion

In recent years, advancement in research has shed some light on the genetic basis and mechanisms of uveitis including VKH disease. Multiple studies have demonstrated that genetic polymorphisms may influence the expression of genes or the functions of gene products and affect the susceptibility of VKH disease (Tables 4 and 5). Some of the major genetic factors involved in immune response associated with uveitis include HLA genes and non-HLA genes such as CTLA-4, ILs, and KIR. The autoimmune response against melanocytes in VKH disease involves a large variety of genes including variants of HLA genes, multiple ILs, and other cytokines and genes involved in the immune pathway. Since each individual gene may have an independent but small effect on the susceptibility and clinical manifestations of VKH disease, this increases the challenge of finding the genetic culprit in pathogenesis of VKH disease.

**Table 4:** Summary of genes that have been shown to be related to increased risk of VKH disease

**Gene**	**Subtype/polymorphism**	**References**
Human leukocyte antigen (HLA)	HLA-DQ4	Islam et al. [[24]]
	HLA-DQw7	Zhang et al. [[25]]
	DR1	Weisz et el. [[30]]
	DR4	Islam et al. [[24]], Zhang et al. [[25]], Weisz et el. [[30]]
	DR53	Islam et al. [[24]]
Cytotoxic T-lymphocyte antigen 4 (*CTLA-4*)	G allele at SNP +49, CTLA-4 haplotype −1661A:−318C:+49G:CT60G	Du et al. [[53]]
Interleukin (IL) genes	*IL-12B* rs3212227 C allele	Li et al. [[67]]
	*IL-17F* rs763780 TT genotype	Shu et al. [[69]]
Killer cell immunoglobulin-like receptors (KIR) gene cluster	*KIR2DS3*, *KIR* gene cluster 3DS1-2DL5-2DS1-2DS5, KIR2DL2/2DL3+HLA-C1	Sheereen et al. [[79]]
Programmed cell death 1 (*PDCD1*)	PD-1.5 C allele	Meng et al. [[98]]
Protein tyrosine phosphatase non-receptor 22 (*PTPN22*)	rs2488457 CC genotype and C allele	Zhang et al. [[107]]
	rs2488457 GG genotype	
Osteopontin (*OPN*)	rs4754 TT genotype	Chu et al. [[110]]
Tumor necrosis factor, alpha-induced protein 3 (*TNFAIP3*)	rs9494885 TC genotype and C allele	Li et al. [[118]]
Macrophage migration inhibitory factor (*MIF*)	rs755622 GG genotype and G allele, rs2096525 T allele, rs755622/rs2096525 CT haplotype	Zhang et al. [[128]]
Fibroblast growth factor receptor 1 oncogene partner (*FGFR1OP*)	rs2301436	Yi et al. [[160]]

**Table 5:** Summary of genes which have been shown to be unrelated or with uncertain association with VKH disease

**Gene**	**Subtype/polymorphism**	**References**
Complement factor H (*CFH*)	184G rs800292 showed increased risk with non-infectious intermediate and posterior uveitis but not in VKH.	Yang et al. [[56]]
Tyrosinase gene family	No significant association in polymorphisms of microsatellites loci in tyrosinase gene family was found in Japanese VKH patients.	Horie et al. [[134]]
Interferon gamma	No significant association in polymorphism of interferon gamma gene was found in Japanese VKH disease.	Horie et al. [[137]]
NLR family, pyrin domain containing 1 (*NLRP1*)	*NLRP1* gene polymorphisms related to vitiligo were not associated with the risks or clinical manifestations of VKH disease.	Horie et al. [[142]]
Toll-like receptor 9 (*TLR9*) gene	No significant association between *TLR9* polymorphisms and VKH disease was found in VKH patients.	Ito et al. [[144]]
Transforming growth factor β receptor (*TGF-*β)	Two SNPs of type III TGF-β receptor, rs1805110 and rs2489188, showed no association with VKH disease.	Chen et al. [[148]]
Janus kinase 1 (*JAK1*) gene	rs310230 GG genotype, rs310236 GG genotype, and rs310241 TT genotype were found in lower frequencies in VKH patients than controls. However, no significant link between the three SNPs and clinical manifestations of VKH disease was identified.	Hu et al. [[150]]
Chemokine (C-C motif) receptor 6 (*CCR6*)	No significant association in *CCR6* SNPs including rs3093024, rs6902119, rs3093023, and rs968334 in Chinese VKH patients.	Yi et al. [[160]]

For future directions of genetics study on VKH disease, in-depth genome-wide scans with fine mapping and exomic sequencing with replications in different ethnic groups will be important for revealing novel genetic regions associated with VKH disease. Detailed studies on correlations of various genotypes and phenotypes of VKH disease should also be carried out. These findings will greatly enhance our understanding in the role of genetic factors in the pathogenesis of VKH disease and will be useful in predicting treatment response and developing more targeted treatment and possible gene therapy.

## Abbreviations

CCR6: chemokine (C-C Motif) receptor 6

CFH: complement factor H

CTLA-4: cytotoxic T-lymphocyte antigen 4

CpG: cytidine-phosphate-guanosine

EAAU: experimental autoimmune uveoretinitis

FGFR1OP: fibroblast growth factor receptor 1 oncogene partner

HLA: human leukocyte antigens

IL: interleukin

JAK1: Janus kinase 1

KIR: killer cell immunoglobulin-like receptors

MIF: macrophage migration inhibitory factor

MHC: major histocompatibility complex

NK: natural killer

NLRP1: NLR family, pyrin domain containing 1

OCA1: oculocutaneous albinism type 1

OPN: osteopontin

PDCD1: programmed cell death 1

PTPN22: protein tyrosine phosphatase non-receptor 22

SLE: systemic lupus erythematosus

SNPs: single-nucleotide polymorphisms

TLR9: toll-like receptor 9

TNFAIP3: tumor necrosis factor alpha-induced protein 3

TGF-β: transforming growth factor β

VKH: Vogt-Koyanagi-Harada

## Competing interests

The authors declare that they have no competing interests.

## Authors' contributions

JN carried out the literature review and drafted the manuscript. FL and TL conceived the review, performed the literature review, and revised the draft manuscript critically. CP conceived the review and revised the draft manuscript critically. All authors read and approved the final manuscript.

## Authors' information

The authors with qualifications are: Joanne YW Ng, MBBS FIona OJ Luk, MRCS Timothy YY Lai, MD, FRCS, FRCOphth Chi-Pui Pang, DPhil.

## Acknowledgements

This paper was presented in part in the 2014 World Ophthalmology Congress, Tokyo, Japan, held in April 2014.
